# A Bottom-Up Approach for Developing Aptasensors for Abused Drugs: Biosensors in Forensics

**DOI:** 10.3390/bios9040118

**Published:** 2019-10-01

**Authors:** Eda Celikbas, Simge Balaban, Serap Evran, Hakan Coskunol, Suna Timur

**Affiliations:** 1 Department of Biochemistry, Institute of Natural and Applied Sciences, Ege University, 35100 Bornova, Izmir, Turkey; simge93balaban@gmail.com; 2 Department of Biochemistry, Faculty of Science, Ege University, 35100 Bornova, Izmir, Turkey; 3 Department of Mental Health and Diseases, Faculty of Medicine, Ege University, 35100 Bornova, Izmir, Turkey; hakan.coskunol@ege.edu.tr; 4 Central Research Testing and Analysis Laboratory Research and Application Center, Ege University, 35100 Bornova, Izmir, Turkey

**Keywords:** point-of-care (POC), custom-tailored aptamers, optical biosensors, electrochemical biosensors, forensic sciences

## Abstract

Aptamer-based point-of-care (POC) diagnostics platforms may be of substantial benefit in forensic analysis as they provide rapid, sensitive, user-friendly, and selective analysis tools for detection. Aptasensors have not yet been adapted commercially. However, the significance of the applications of aptasensors in the literature exceeded their potential. Herein, in this review, a bottom-up approach is followed to describe the aptasensor development and application procedure, starting from the synthesis of the corresponding aptamer sequence for the selected analyte to creating a smart surface for the sensitive detection of the molecule of interest. Optical and electrochemical biosensing platforms, which are designed with aptamers as recognition molecules, detecting abused drugs are critically reviewed, and existing and possible applications of different designs are discussed. Several potential disciplines in which aptamer-based biosensing technology can be of greatest value, including forensic drug analysis and biological evidence, are then highlighted to encourage researchers to focus on developing aptasensors in these specific areas.

## 1. Introduction

In forensic sciences, the extensive spectrum of analytes and sample types which may range from simple visuals (e.g., fingermarks) to complex biological molecules (e.g., DNA) are used [1]. Abused drug analysis may be considered as one of the most paramount branches in forensics. Various analytical methods are used to determine the presence of one or several compounds as an abused drug in different biological matrices. To date, complex analytical systems, such as high-performance liquid chromatography (HPLC) [2], capillary electrophoresis (CE) chromatographic systems [3], gas chromatography–mass spectrometry (GC/MS) [4], liquid chromatography tandem mass spectrometry (LC-MS/MS) [5], have been used for forensic drug analyses [6]. However, these high technology devices are complex and costly methods that require expensive equipment and an expert person to conduct the analysis. A continuing change in the area of medical diagnostics is created by the development of biosensors that can enable the analysis to be conducted at point-of-care (POC).

A biosensor is an analytical device that combines a recognition element with a transducer to produce a measurable signal that can be correlated with the concentration of the analyte of interest [7]. An ideal biosensor is defined as affordable, sensitive and specific, user-friendly, rapid and robust, equipment-free, and deliverable to those in need, namely ASSURED, by the World Health Organization (WHO) [8]. For a POC diagnostics device to become commercialized, conventional biological test formats should be minimized to the size of a handheld device using a small amount of sample and reagents, while providing a multi-analyte, high-throughput, specific and sensitive analysis [7]. POC diagnostics work with small samples volume, respond within seconds, enable on-site and equipment-free diagnostic, enable cost affordable, specific and sensitive detection [9]. These tests are designed to give both quantitative and qualitative results obtained from the interaction between analytes and target-specific recognition agents. The recognition elements can be antibodies, aptamers, or enzymes in different designs. Mostly used POC methods include optical (colorimetric, fluorescence-based, surface plasmon resonance (SPR), etc.) and electrochemical measurements that can be used for interaction monitoring [10,11,12].

Until recently, many of the methods used in POC diagnostics in forensic science only included immunoassay-based biosensors which used antibody–antigen interactions [13]. With the advancements in aptamer development technologies, aptasensors have become more widely used due to the advantages they possess. Aptamers are short, single-stranded DNA or RNA sequences that are able to undergo selective antigen association as a result of three-dimensional structure formation [14]. Aptamers have many convenient properties, such as high stability, long shelf-life, being easily producible and modifiable, easily transportable and storable, low immunogenicity, and variety of targets [15]. Thanks to these properties, different analytes are detected in low concentrations with aptamer-based POC platforms [16,17,18,19], and aptasensors may overthrow immunoassay-based biosensors in forensics in the near future.

Despite their immense potential in biological diagnostics at POC, aptamer-based biosensors have yet to find their place in forensic sciences for number of reasons including very few aptamer sequences have been developed for relevant targets [20], the need for successful real-life samples application [13], and already-spent money and facilities for antibody-based detection techniques in analytical laboratories [21,22]. In this review, a bottom-up approach for developing aptasensors in the field of forensics is followed by describing the recent technologies used in custom-tailored aptamer development strategies for relevant analytes and presenting their applications in biosensors for the determination of abused drugs. This review attempts to enlighten the current state of the aptasensor device designs in the forensics field and highlights some important biosensor formats that have recently been applied to abused drug detection emphasizing the advantages or disadvantages of each biosensor type with respect to its applicability in the POC diagnostics devices.

## 2. Custom-Tailored Aptamer Development

### 2.1. Principles of Aptamer Selection

**Figure 1:**
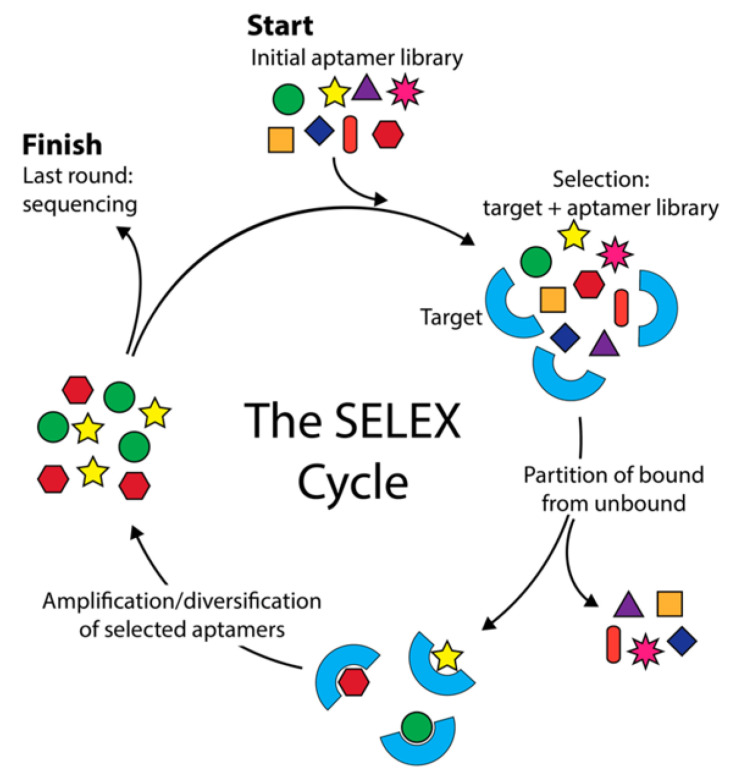
The systematic evolution of ligands by exponential enrichment (SELEX) cycle. SELEX starts with a random nucleic acid aptamer library which is used to initiate the SELEX cycle (top arrow entering cycle). The library is incubated with the target, and the target is washed to remove and discard unbound aptamers (right arrow exiting cycle) before the bound aptamers are eluted and amplified by PCR. The amplified sequences seed the next round of SELEX. Typically, around 12 SELEX cycles are performed before sequencing and aptamer characterization (left arrow exiting cycle). Reproduced with permission of [33]. Copyright 2017 Published by MDPI.

One major advantage of aptamers over antibodies is that aptamers can be developed in vitro by systematic evolution of ligands by exponential enrichment (SELEX). In vitro selection of aptamers is particularly significant for small molecule targets. Abused drugs show weak immunoreactivity due to their small molecular size. Hence, the design and the choice of carrier proteins become critical for the success of antibody production [23]. In contrast, the SELEX process basically relies on the selection of target-binding sequences from a random oligonucleotide library (Figure 1). The initial nucleic acid pool consists of ~ 10^14^ to 10^16^ single-stranded DNA (ssDNA) or RNA molecules containing a central random sequence flanked by two constant primer binding sites at 5′ and 3′ ends. SELEX process includes iterative steps of binding, elution, and amplification. The target of interest is incubated with the random library under predetermined conditions. Then, unbound sequences are removed and discarded. The bound sequences are eluted from the target, amplified, and used in the next round. Incorporation of negative-SELEX and counter-SELEX enhances the specificity of aptamers. Negative-SELEX is performed for the elimination of non-specific sequences that bind to the immobilization matrix. Counter-SELEX is similar to negative-SELEX, except that the counter target is a molecule that is structurally similar to the target. SELEX is repeated for several rounds until the high-affinity binders are enriched. Finally, the resulting pool is sequenced, and the selected aptamers are characterized for their binding affinity. Since the first introduction of the SELEX method in 1990 by two independent groups [24,25], a large number of aptamers have been developed for various targets ranging from small molecules [26,27] to proteins [28,29], whole cells [30], and tissues [31,32].

### 2.2. Experimental Design of SELEX

SELEX technology has proved to be applicable to small molecule targets, albeit with some challenges [34,35]. Covalent immobilization of the small-molecule target to a solid support (e.g., magnetic beads) facilitates partitioning of bound and unbound sequences during SELEX [36,37,38]. However, the chemical coupling of the small-molecule target through its functional group may severely affect aptamer–target interactions [39]. Thus, the Capture-SELEX [40,41,42] and Graphene oxide-assisted SELEX (GO-SELEX) [43,44] methods have been developed to eliminate the target immobilization. Both methods have been successfully used to develop aptamers for a number of small molecules [45,46,47,48].

Although the basic concept of SELEX is the same, there are several experimental parameters that may affect the overall success for each target [49]. One of the crucial points is the design of the initial nucleic acid library [50]. Single-stranded DNA or RNA libraries can be chemically synthesized and amplified by polymerase chain reaction (PCR). RNA aptamers can be genetically encoded in cells, which allows the design of intracellular sensors [51]. DNA aptamers present advantages in terms of cost, ease of synthesis, and stability. Another issue is the length of the randomized region. Short randomized regions ensure representation of each sequence in the pool, albeit with less structural complexity. Although longer libraries do not cover the full sequence space, the possibility to form more complex structural motifs increases [52]. To impart additional physicochemical diversity to aptamers, nucleobase modifications can be introduced to the library [53]. Slow off-rate modified aptamer (SOMAmer) technology has been developed by the SomaLogic company to impart protein-like hydrophobic interaction ability [54]. Click-SELEX is also an elegant approach that relies on chemical modification of the library via click chemistry [55]. In recent years, computational approaches have also been used to increase structural complexity or to explore the sequence space [33,56,57,58].

Binding affinity of aptamers is expressed as dissociation constant (K_d_) that can be determined by various techniques, such as surface plasmon resonance (SPR) [59], isothermal titration calorimetry (ITC) [60], fluorescence spectroscopy [61,62], equilibrium dialysis [63], backscattering interferometry (BSI) [64], enzyme-linked oligonucleotide assay (ELONA) [65], and combined ELONA real-time quantitative PCR (qPCR) [66]. Post-SELEX optimization via truncation, chemical modification of nucleobases, or mutagenesis can be performed to improve the binding affinity or sensor properties [67]. The secondary structure of the aptamer can be computationally predicted by using Mfold [68], RNAstructure [69], and ValFold [70]. Circular dichroism (CiD) [71], nuclear magnetic resonance (NMR) [72], X-ray crystallography [73] as well as molecular modeling [74] are useful to probe aptamer–target interactions. Using the structural information, the full-length aptamer can be subjected to post-SELEX to obtain the optimal sequence [75,76,77,78,79,80].

### 2.3. Selection and Characterization of Aptamers Against Abused Drugs

Forensic science has benefited from SELEX technology, and great progress has been made to design aptasensors for forensic analysis [22]. As summarized in Table S1, DNA or RNA aptamers have been developed against a few abused drugs. Among them, the cocaine aptamer is the most extensively studied one for its ligand binding properties. First, the DNA aptamer MNS-4.1 for cocaine was introduced and then engineered to aptamer MNS-7.9 to design a fluorescent sensor [81]. The binding pocket of MNS-4.1 was proposed to be localized in the lipophilic cavity of a three-way junction. Moreover, the short S3 stem was proposed to form only in the presence of a ligand. The authors attempted to engineer instability by shortening the S1 stem and successfully obtained aptamer 7.9. However, the binding affinity of the constructed 7.9 aptamer was found to be lower compared to the original aptamer. The F7.9D aptamer bearing 5′-fluorescein and 3′-dabcyl was shown to be selective over the metabolites of cocaine, namely benzoyl ecgonine and ecgonine methyl ester. Binding experiments proved that F7.9D lacks significant enantiodiferentiation. The authors concluded that both (−) and (+) cocaine could induce folding of the aptamer, whereas the metabolites lacking a stabilizing group or possessing a destabilizing group interfere with the formation of the highly negatively charged three-way junction.

Following initial characterization of the cocaine aptamer, four sequence variants were constructed by either lengthening or shortening the stem 1. The authors concluded that the length of the stem containing 3′ and 5′ termini has a significant role in the binding mechanism [82]. In a similar study, it was revealed that the shortening of each of the three stems or disrupting specific base pairs at the three-way junction results in reduced affinity [83].

Further research focused on the binding promiscuity of the cocaine aptamer. The MN4 aptamer with a longer stem 1 and the MN19 aptamer with a shorter stem 1 have been shown to bind the off-target ligand quinine 30 to 40 times stronger than cocaine. Based on the structural comparison of MN4 and MN19, many similarities and some differences were found in the binding mechanism of cocaine and quinine [84]. The binding affinity of MN4 was tested against thirteen quinine analogs, as well as a set of cocaine metabolites and the alkaloid atropine. It was concluded that the aromatic region of the ligand, the substituents on the aromatic ring, and the stereogenic center at carbon 9 are the key factors that affect binding [85]. In another study, the MNS-4.1 aptamer was also shown to bind the fluorescent dye 2-amino-5,6,7-trimethyl-1,8-naphthyridine (ATMND) [86]. The Cocaine-binding aptamer was shown to bind cocaine, norcocaine, cocaethylene, and 6,7-dehydronorcocaine with affinities in the range of 10 to 56 μM. Having shown that the cocaine aptamer is sensitive to NaCl, the authors proposed selective 2-aminopurine (2AP) substitution as a novel way to eliminate the effect of NaCl and the matrix effect, thereby improving the binding specificity of cocaine aptamer [87]. In another study, it was shown that the cocaine-binding aptamer switches from one-site to two-site ligand binding, depending on NaCl concentration [88]. Pulsed electron–electron double resonance (PELDOR) spectroscopy was used to monitor the conformational change of cocaine aptamer [89].

SELEX technology has enabled the selection of RNA aptamers for codeine [90]. First, codeine was immobilized on epoxy-activated agarose through its hydroxyl group and then incubated with an RNA library containing a 30 nucleotide random region. The binding buffer was composed of 250 mM NaCl, 20 mM Tris-HCl (pH 7.4), and 5.0 mM MgCl_2_. The two high-affinity aptamers, namely FC5 and FC45, were selected for further structural analysis. The authors showed that the mini-aptamers FC5L and FC45L display similar binding affinity compared to the full-length aptamers. The importance of the formation of the correct binding pocket was revealed after binding experiments and secondary structure predictions of the modified forms of mini-aptamers. Both FC5 and FC45 was shown to be highly selective to codeine over morphine. Interestingly, FC5 was found to display higher specificity to thebaine, which is structurally similar to codeine. 

This study was followed by a second SELEX study, and the DNA aptamer was developed for codeine [91]. Similar to the previous study, codeine was immobilized on epoxy-activated agarose and then incubated with an ssDNA library containing a 40 nucleotide random region. However, a different binding buffer that is composed of 140 mM NaCl, 2.5 mM MgCl_2_, 2.5 mM KCl, 1.6 mM KH_2_PO_4_, 15 mM Na_2_HPO_4_, and 0.02% tween 20 (pH 7.4) was used. The aptamer named HL7-1 was obtained and subjected to several truncation–mutation experiments. Finally, HL7-14 was optimized and used for the construction of an electrochemical biosensor. The binding properties of HL7-14 have been shown to be affected by Na^+^ and K^+^ ions, as well as pH. HL7-14 has been found to be highly selective to codeine over morphine and several amino acids.

Methamphetamine was also targeted to develop a DNA aptamer [92]. Methamphetamine was immobilized on epoxy-activated agarose and then incubated with an ssDNA library containing a 40 nucleotide random region. The binding buffer was composed of 200 mM NaCl, 20 mM Tris-HCl (pH 7.4), and 5.0 mM MgCl_2_. A counter-SELEX step with amphetamine was incorporated to increase the specificity for methamphetamine. After a total of 14 SELEX rounds, 29 clones were sequenced, and the aptaMETH with a K_d_ value of ~100 nM was identified. AptaMETH has been shown to be highly selective to methamphetamine over the structurally similar amphetamine molecule.

Ephedrine aptamer was recently selected by using the GO-SELEX method [93]. First, an ssDNA library with a random sequence of 40 nucleotides was prepared by using the long and short-chain method. To ensure binding specificity over pseudoephedrine, counter-SELEX was performed from the 6th round to the 10th round. In this study, resonance Rayleigh scattering (RRS) technology was used for the first time to monitor the screening process and determination of binding affinity. The aptamer named EP08 displays strong binding and significant selectivity was obtained.

## 3. Aptasensor Development

### 3.1. Advantages of Aptamers in Biosensor Development

**Figure 2:**
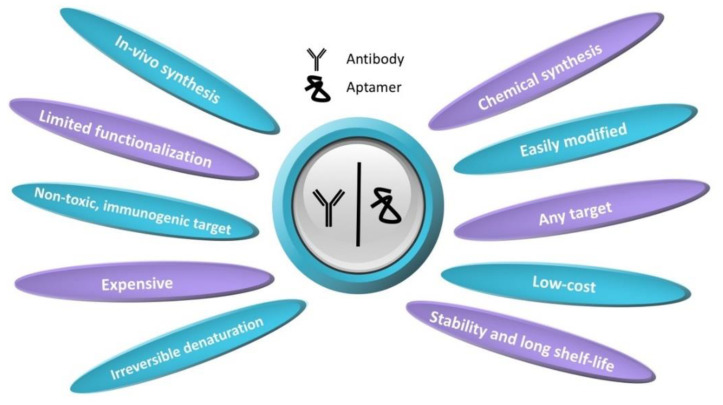
Main advantages of aptamers compared to antibodies.

The use of aptamers in biological sensing platforms is considered to be advantageous due to several useful properties they possess over antibodies to replace the recognition element of the sensor [94,95]. The main advantage of the aptamers is the elimination of the use of animal experiments for the production. Antibody production mostly involves stimulation of the immune response to the target analyte, but the immune system may fail when the target molecule has a familiar structure with the endogenous proteins. Whereas aptamers are procured from in vitro methods that do not include animals. Additionally, antibodies are restricted by physiological conditions when recognizing the target, and this limits the functionalization and application of the antibodies. However, the aptamer selection process can be easily modified to obtain correct forms of the aptamers that can recognize a specific region of the target in different binding conditions [95]. In addition, it was noted that the studies with antibodies contained deficiencies in quality and reproducibility, that less than half of the antibodies could be verified to recognize their targets [96]. Commercially produced antibodies are known to exhibit poor specificity for target molecules or are insufficient to recognize such molecules. Whereas aptamers are RNA-based, and they have a more flexible backbone, small, stable, and a wide range of potential target molecules [97]. After the selection of the sequence, aptamers can be synthesized chemically at a high purity rate, and modifications can be easily applied to enhance stability, affinity, and specificity of the molecules. As they have a simple structure, the layer-by-layer coatings can be formed easily. In addition, the structure of antibodies is large multidomain protein complexes containing specific disulfide bonds and glycosylation, and antibody production is often difficult and expensive compared to aptamer production [98]. Last but not least, aptamers are resistant to denaturation and have longer shelf-life. Main advantages of aptamers over antibodies are summarized in Figure 2. Due to these favorable properties, aptamers have been widely used in the POC diagnostics in the last two decades.

According to the International Union of Pure and Applied Chemistry (IUPAC) definition “a biosensor is a self-contained integrated device composed of a bio-recognition element that is coupled to a physicochemical detector element or transducer which converts the recognition event into quantitative or semi-quantitative analytical information” [99]. Biosensors offer an alternative to conventional analytical devices, such as spectrophotometry or HPLC, as the advanced devices limit the continuous monitoring. They are not easy to operate; they need well-trained operators and require additional sample preparation steps which decrease the time-efficiency and cost-effectiveness of the analysis. Biosensors offer a wide range of application due to their specificity, simplicity, low-cost, rapid response, and the enablement of continuous monitoring [100]. Basically, a biosensor consists of two essential components: target recognition and signal transduction. A biosensor that uses aptamers as a target recognition agent is called an ‘aptasensor’ [101]. As signal transduction, several different methods including electrochemical [102,103,104], optical [81,105,106], mass-sensitive transduction modes (i.e., SPR-based techniques) [107,108] have been proposed to date for aptasensors. However, optical and electrochemical methods have been more widely used for the detection of abused drugs in recent literature.

### 3.2. Immobilization Techniques of Aptamers for Aptasensors

Analytical diagnostic platforms generally require the immobilization of the recognition element onto a solid substrate, unless a solution-based approach is followed. The main concern in selecting the correct immobilization method is maintaining the binding affinity and selectivity that aptamer displays in the solution phase while protecting the sensitivity of the optical transducer [109]. This suggests that the chosen technique should provide low optical absorption, high-density packing and binding efficiency, specificity, and stability in the testing media and create a surface that does not easily damage and can be used several times [110]. There are several methods established for immobilization of the aptamers, and all are rooted from the same principles applied for the immobilization of DNA-based biomolecules [111]. Exploited in the literature, according to the abundancy, the techniques used for immobilization is classified as follows: direct attachment to gold, covalent attachment to functionalized surfaces, and bio coatings [109,110,112,113]. Direct attachment to gold substrates (e.g., electrodes, nanoparticles) is performed via chemisorption of thiol modified aptamers onto gold and has many advantages, such as the ease of immobilization, the ability to form a monolayer, the stability of the gold surfaces, high affinity to thiol-modified substances, and commercial availability [114]. However, the attachment to the surface is relatively weak, limited by gold substrates and the orientation of the recognition molecule is random. The covalent attachment can be formed via the interaction between functionalized aptamers and the corresponding chemical groups on the immobilization surface with the appropriate cross-linkers [113]. This method has the advantages of wide application range, increased specificity, and decreased interference signals, however, still suffers from complex modification steps and non-specific binding. The bio coatings approach refers to the strong and specific interaction between biotin and streptavidin, which is fairly exploited in the literature using aptamers [115,116]. Compared to other methods, as each streptavidin molecule can bind four biotinylated aptamers, aptamer binding on the surface is increased, reducing the nonspecific adsorptions and improving sensor signal-to-noise ratio [113]. However, the pH values have a considerable effect on streptavidin chemistry which has the potential to facilitate an undesired reaction between aptamers and streptavidin, causing a mislead on the signals. Being one of the key steps in aptasensor development, immobilization of the aptamers will not only affect the properties of aptamers but also influences the performance and accuracy of the results [113].

### 3.3. Optical Aptasensors

**Figure 3:**
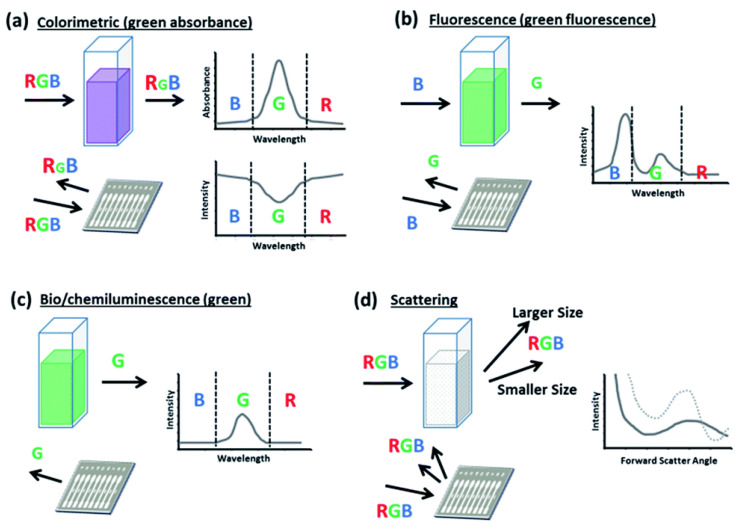
Typical optical detection for both light transmitted through liquid sample platforms (i.e., cuvette, well plate) and light reflected from solid sample platforms (i.e., test strip, cassette) using (**a**) colorimetric assays, (**b**) fluorescence-based assays, (**c**) bio- and chemiluminescence assays, and (**d**) scattering-based assays. Reproduced with the permission of [106]. Copyright 2016 Published by The Royal Society of Chemistry.

Due to their high performance in terms of sensitivity, selectivity, response time, and operational simplicity, optical aptasensors have been widely used for the detection of abused drugs. Optical biosensors typically use four different signal detection principles: colorimetric, fluorescence-based, chemi-/bioluminescence-based, and scattering-based sensing (Figure 3) [117,118]. These systems can be formed in two formats which are solution-based and flat substrate-based assays. The literature survey of the detection of abused drugs is abundant of optical biosensors, using colorimetric [119,120,121,122,123,124,125,126,127], fluorescence-based [86,128,129,130,131,132], chemiluminescence-based [133,134,135], and SPR-based techniques [136]. The results from the literature survey are summarized in Table S2.

#### 3.3.1. Colorimetric Aptasensors for Abused Drugs

Colorimetry is one of the simplest sensing phenomena, and hence, is explored in many different studies in the literature [137,138]. In its most basic form, colorimetric sensors measure the changes in the intensity of absorbed or reflected light emitted from the conjugates in response to the recognition of element–target binding events [139]. These events are mostly monitored with an optical property change over a wide range due to surface plasmon resonance or structural shifts in organic dyes, luminophores, conjugated polymers, or metal nanoparticles and nanostructures [118,140,141]. Gold nanoparticles (AuNPs) and their core–shell variations have been used tremendously for the colorimetric biosensors due to their many beneficial properties including the simplicity of the synthesis, surface modifiability, biocompatibility, and being mostly colored to provide visibility even to the naked eye, practicality, and efficiency in analysis [142]. Salt induction of AuNPs, which refers to the decrease in the screening length of charged chemical groups on the nanoparticle surface and inducing the instantaneous and irreversible aggregation of the nanoparticles using a high concentration of salts, is a widely used colorimetric screening method [143,144]. Optical readouts can provide qualitative and/or quantitative results for the color change with a simple spectrophotometer or more popularly, with a smartphone and an image analysis software [145,146]. Several softwares have been reported in the literature for practical analysis of the images for colorimetric sensing, including Image J, Fiji, The Image Studio, and Image Lab [147,148]. This detection technique enables on-site and point-of-care measurements to be simple, rapid, and low cost, using either no or user-friendly equipment.

Since 2000, colorimetric aptasensors have been widely researched and exploited by the POC diagnostics researchers. However, the literature on colorimetric aptasensors for abused drugs is not very fruitful, to the best of our knowledge, only eight studies were conducted in this field. AuNPs [119,120,123,125,127,149,150], Au@Ag core–shell nanoparticles [124], and 4, 4’-diamino-3, 3’, 5, 5’-tetramethylbiphenyl (TMB) [121,122,126] chemistry were used as the colorimetric agents. Mostly, simple Eppendorf or µ-well-based techniques were established and used for the detection of cocaine and methamphetamine [121,122,123,125,126,127,149,150]. These studies either screened catalytic activity of horseradish peroxidase (HRP) for the HRP–TMB–H_2_O_2_ colorimetric system [121,126], used hemin chemistry to create a colorimetric probe [122] or salt-induced aggregation principle of AuNPs [123,125,127,149,150]. UV-Vis absorbance spectra were used to conduct the analysis for all the studies. 

The main disadvantage of salt-induced aggregation of AuNPs is the lack of efficiency in the detection of lower concentrations of analytes which is limited by the aggregation-induced sedimentation [151]. Hence, to increase the sensitivity of the assay, Mao et al. developed a novel colorimetric sensor based on a non-aggregated Au@Ag core–shell nanoparticles for the detection of cocaine and methamphetamine [124]. Three different probes were used for the detection: reporter probe (RP) which was the Au@Ag core–shell nanoparticle modified with a RP DNA, capture probe (CP) which was carboxyl-coated magnetic beads (MBs) conjugated with a CP DNA, and an aptamer which would bind to RP and CP, forming an Au@Ag-DNA-MBs sandwich structure. Applying an external magnetic field, in the absence of the target analytes, the sandwich structure was removed from the solution, reducing the intensity of SPR signal from the Au@Ag core–shell nanoparticles. When the analytes were found in the solution, aptamer binding to the two probes is prevented, and the aptamer was bound to the analyte preferably. Thus, the concentration of Au@Ag nanoparticles in the solution was found to be proportional to the concentration of the target drugs in the solution. In this study, methamphetamine and cocaine were successfully detected with a limit of detection (LOD) of 0.1 nM and 0.5 nM, respectively. Additional studies with interfering molecules and spiked urine sample analysis showed promising results, proving the value of the study.

Lateral flow assays (LFAs) became one of the dominant technologies in POC diagnostics which use colorimetric sensing strategies as they provide qualitative, semi-quantitative, and quantitative measurements in resource-limited settings and non-laboratory environments. The LFAs are mainly created with a sample pad, conjugate pad, analytical membrane, absorbent pad, and a backing pad. When the sample is applied on the sample pad, the sample migrates along the conjugate pad, carrying the antibody modified labeling particles (e.g., AuNPs) through the analytical membrane towards the absorbent pad via a capillary force provided by the absorbent pad. While passing towards the test zone, which comprises the immobilized capturing molecules (e.g., antibodies), target analytes interact with the recognition element, and a test line is formed. There are two different formats of LFAs: sandwich and competitive format [152]. In the sandwich format, the recognition molecules labeled with a color providing agent interacts with the analyte, and this conjugate is then captured on the test line with the corresponding capturing molecules [153,154]. In the competitive format, the recognizing element reacts with the capturing molecules on the test zone, and the analyte of interest competes with the recognition element for binding [155,156]. The sandwich format is used when there are multiple epitopes in the target analytes, while the competitive format is used when the target analytes are molecular weight or have a single specific antigen [157]. LFAs provide low-cost, rapid, portable, and modifiable tools in POC diagnostics.

**Figure 4:**
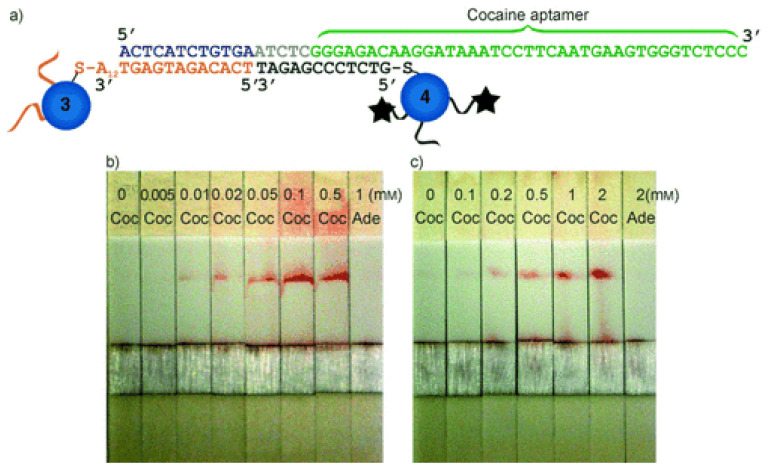
Lateral flow based detection of cocaine. (**a**) DNA sequences and linkages in cocaine aptamer-linked nanoparticle aggregates. Test of the cocaine-sensing lateral flow device with varying concentrations of cocaine in buffer solution (**b**) and in undiluted human blood serum (**c**). Coc = cocaine, Ade = adenosine. Reproduced with the permission of [109]. Copyright 2006 Published by Wiley-VCH.

Many efforts have been made to increase the sensitivity, reproducibility, quantification, and multiplexing capability of LFAs [157]. Mostly antibodies are used as recognition molecules in the literature [12,158]. However, aptamers are not yet adapted, especially not in the abused drug detection. Only one study has been reported in the field of LFAs using aptamers as recognition molecules for cocaine analysis [120]. In this study, LFA consisted of the common four types of membranes. The cocaine aptamer conjugated nanoparticle aggregates were spotted on the conjugation pad, and streptavidin was applied on the analytical membrane as the test line. The hypothesis was that the nanoparticle aggregates were large enough to not be able to flow throughout the membrane, whereas dispersed nanoparticles can reach to the end of the stick. When dipped into the solution, in the absence of the analyte, aggregates could be rehydrated and move along the strip, stopping at the bottom because of their large size. However, in the presence of the analyte, nanoparticle aggregates would disassemble due to the interactions between the analyte and aptamer. Thus, re-dispersed nanoparticles could then move along the membrane and form the res line on the test zone, in correlation with the concentration of the analyte. With this method (Figure 4), cocaine measurement was conducted with an LOD of 10 µM, and spiked serum samples were also successfully analyzed.

#### 3.3.2. Fluorescence-based Aptasensors for Abused Drugs

Fluorescence has been applied to different biosensing systems, as well as aptasensors, due to its advantageous properties, such as high sensitivity, high efficiency, and simple operational steps. Fluorescence-based analytical platforms measure the light emitted from the target sample due to a radiative excitation. A narrow peak of emission wavelengths provides measurements in lower concentrations and can be tuned to increase the sensitivity even more with changing excitation source intensity [139]. There are two main modes of fluorescence-based techniques: labeled and label-free biosensing systems. Naturally, only a few biomolecules and aptamers are autofluorescence, and thus, a label is crucial to obtain a measurable signal [118]. 

Most commonly used fluorescence labels are small molecule fluorophores, quantum dots (QDs), and fluorescent nanoparticles [159]. Small molecule fluorophores are molecules that are mostly water-soluble, low molecular weight (below 1 kDa), and usually have reactive functional groups that can provide conjugation with recognition elements, such as antibodies, aptamers or peptides [160]. Due to their low molecular weights, properties of conjugates of small molecule fluorophores with larger biomolecules tend to be similar to the attached biomolecule (e.g., antibodies) rather than the fluorophore [161]. QDs are defined as semiconductor nanoparticles that emit fluorescence when excited with radiation [139]. They are usually either encapsulated or conjugated with functional polymers as they lack water solubility and biocompatibility [160]. Compared to other fluorescence agents, nanoparticles are mostly biocompatible, have photostability, and rarely get affected by the nonspecific interactions in terms of their optical and structural properties [162]. Nanoparticles are good quenchers of fluorescence and are usually applied in a fluorescence resonance energy transfer (FRET) mechanism [162,163].

The major advantages of fluorescence-based POC diagnostic platforms compared with colorimetric sensing methods are simplicity, high selectivity and sensitivity, low detection limits, fast response time, and ease of handling [164]. However, fluorescence-based sensors suffer from background excitation light interference, which may mislead the accuracy of the experiments. Significant efforts have been made to develop instruments that can be facilitated to eliminate such signals to improve fluorescence-based techniques [165,166].

**Figure 5:**
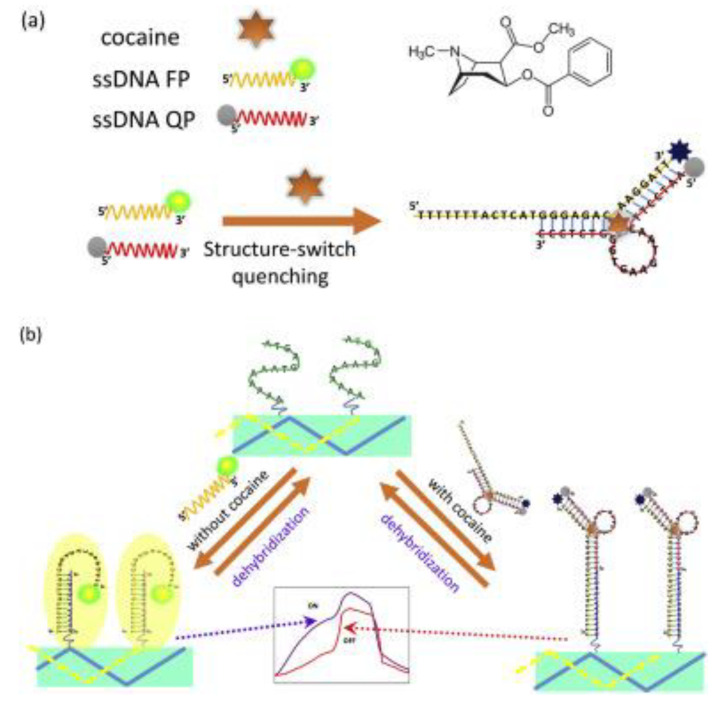
Schematic of cocaine sensing based on structure switching optical biosensing platform. (**a**) Structure switching of cocaine combining the two aptamer fragments; (**b**) hybridization and regeneration of the aptamer with optical fiber. Reproduced with the permission of [119]. Copyright 2016 Published by Elsevier B.V.

In the field of abused drug aptasensors, fluorescence-based optical aptasensors are mainly focused on labeled modes of detection for cocaine [128,129,130,131] and methamphetamine [132] analysis. As labels, carbon dots (CDs) [132] and fluorescent dyes (pyrene [129], fluorescein (FAM) [128], SYBR Green I [131] and Cy3 [130]) were used and facilitated with FRET measurements [132] and fluorescence quenching [128]. One on-demand strategy that was mostly used was strand displacement of aptamers in the presence of target molecules [129,130,131]. This molecular engineering strategy involves using more than one labeled, target-induced complementary aptamer fragments instead of a single strand aptamer sequence while maintaining its affinity to the target molecule. The conformational structures of the fragments were switched when the analyte was introduced to the solution enabling the formation of an aptamer–target complex, resulting in a change in the fluorescence characteristics of the system. This strategy provides high selectivity and sensitivity for simple, rapid, and real-time monitoring of biomolecules in various biological samples and is expected to find its place in the literature in the fields of clinical diagnosis, biological analysis, and drug screening [129]. The principles of detection for this platform is presented in Figure 5.

An aptasensor design that used label-free transduction methods was reported by Roncancio et al., enabling the use of unmodified samples via an auto-facilitating aptamer-fluorophore assembly [86]. In this study, it was found that the cocaine-binding aptamer, MNS-4.1, can also bind the fluorescent molecule 2-amino-5,6,7-trimethyl-1,8-naphthyridine (ATMND) while quenching its fluorescence. In the presence of target analyte, cocaine, in this case, ATMND was competitively replaced by cocaine from the aptamer recognition site, and a high fluorescence signal was measured as the ATMND was released into the solution. Using this highly sensitive method of screening, cocaine was detected with an LOD of 200 nM within 20 s. Furthermore, this sensing strategy was also successful in measuring cocaine in biological fluids in the micromolar range. 

#### 3.3.3. Other Optical Aptasensors for Abused Drugs

In addition to colorimetric and fluorescence-based aptasensors, chemiluminescence and SPR-based techniques were also used for abused drug detection. Different from other optical detection methods, in chemiluminescence, excitation energy comes from the chemical reaction itself. As the luminescence signal, which can be measured down to only a few emitted photons, is a highly detectable bio- or chemiluminescence technique, it has become favorable [167]. High sensitivity, wide calibration ranges, and simple instrumentation comprise the unique properties of chemiluminescence-based biosensor platforms. Several chemiluminescence-based aptasensors have been successfully applied to abused drug sensing [133,134,135]. All three studies were focused on the detection of cocaine. Yan et al. [134] used HRP, and alkaline phosphatase (ALP) to label different reporter DNA probes, and catalyzed chemiluminescence reactions were monitored. A relatively low LOD value of 3.2 nM was achieved with this straightforward strategy, proving its worth to be implemented in future studies for small molecules. A similar approach was used by Li et al. [135], using the chemiluminescence signals of luminol-H_2_O_2_-HRP-*p*-iodophenol (PIP) system. AuNPs were modified with magnetic microbeads (MB-AuNPs), and aptamers were immobilized on the surface of this conjugate. The complex was then hybridized with the signal DNA modified with HRP and in the presence of the cocaine, analyte and signal DNA were competed, forcing the MB-AuNPs to dissociate due to the aptamer structure switching. Thus, the chemiluminescence signal was obtained proportional to the cocaine present in the solution. With this method, 0.48 nM LOD value was obtained successfully.

Additionally, electrogenerated chemiluminescence (ECL) approach was facilitated by Sun et al. [133], focusing on the detection of cocaine. ECL is a technique where the light is generated by highly reactive species via electrochemical reactions on the electrode surface [168,169]. ECL has many beneficial properties, such as high sensitivity and selectivity, reproducibility, and low background, which facilitated this method use in many different chemical and biochemical applications [170]. In work by Sun et al., an oxidative-reduction type Ru(bpy)_3_^2+^ (Ru1, or its derivatives)/tri-*n*-propylamine (TPrA) system was combined with aptasensing technology which provided high-efficiency ECL emissions in aqueous solutions. ECL emissions from the chemical sensor were produced via cyclic voltammetry (CV) scans. A significantly sensitive, electrochemically stable, and highly reusable ECL sensor was developed and very low LOD values for cocaine, such as 10 pM, were achieved with this technique.

**Figure 6:**
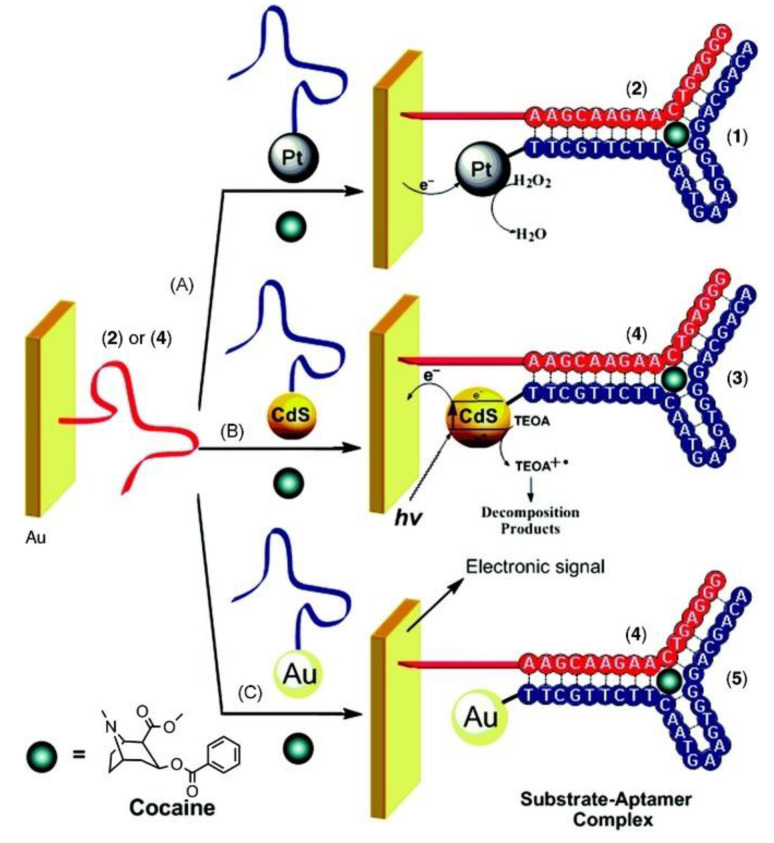
Electrochemical (Path A), photoelectrochemical (Path B), and surface plasmon resonance (SPR) (Path C) analysis of cocaine. Reproduced with the permission of [125]. Copyright 2009 Published by American Chemical Society.

SPR is an optical–electrical technology based on the change in the refractive index due to the interaction of light with the electrons of a metal surface which is directly proportional to the mass of the biomolecule thickness or mass on the surface [7,118]. This method provides rapid, label-free, real-time monitoring with a small sample amount [7]. Even though SPR has been thoroughly used for biological samples [171,172], to detect small molecules with this method still remains as a challenge as the aptamer molecule causes a too small change in the refractive index for analysis. Thus, aptasensors adapting SPR as an analytical technique usually use metal nanostructures as a signal enhancement tool [140,173]. In work conducted by Golub et al. [136], electrochemical, photoelectrochemical, and SPR detection of cocaine was successfully carried out using two cocaine aptamer subunits. One subunit is attached to an SPR-active Au surface, and the other subunit is labeled with either platinum nanoparticles (PtNPs), CdSNPs, or AuNPs, depending on the method to be used. AuNPs were used for SPR-based detection mode (Figure 6). In the presence of cocaine, a supramolecular complex was formed between the AuNP-cocaine aptamer and the cocaine on the Au surface, enabling the SPR monitoring of cocaine according to the reflectance changes resulting from the electronic coupling between the localized plasmon of the Au-NPs and the surface plasmon wave. A low range of LOD values, 1 to 10 nM, was achieved using this technique.

### 3.4. Electrochemical Aptasensors

**Figure 7:**
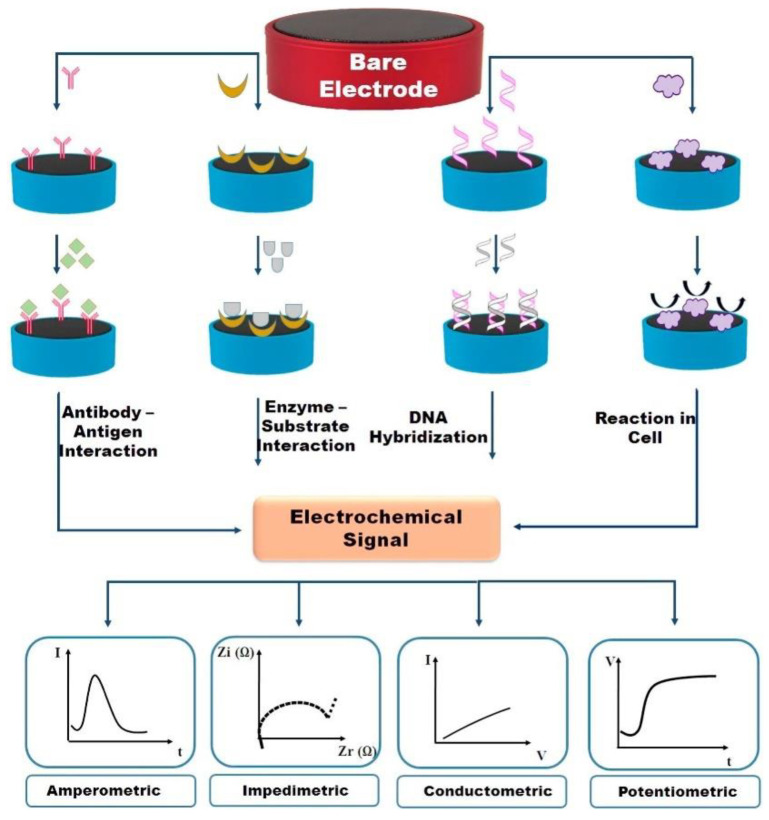
Typical electrochemical biosensor measurement principles. Reproduced with the permission of [166]. Copyright 2015 Published by Elsevier B.V.

Electrochemical aptasensors have been one of the most widely used aptasensor classes for detection of abused drugs due to beneficial properties, such as low-cost, rapid response, working with a minimum sample, high sensitivity and selectivity, and enabling fieldwork with commercially available battery-powered equipment [174]. Electrochemical sensor systems are defined as a molecular detection device which converts the information received from a biological recognition element into an electrical signal using electrodes via a transducer [175]. These systems are generally formed with a three-electrode system which includes a reference electrode that is commonly made from Ag/AgCl, a working electrode that can be either carbon, gold or graphene-made, and a counter or auxiliary electrode, which generally is a Pt electrode [176]. Electrochemical biosensor platforms can be divided into four major categories in terms of their measurement principles: amperometric, impedimetric, conductometric, and potentiometric (Figure 7) [177]. However, amperometric [11,91,178,179,180,181,182,183,184,185,186,187,188,189,190,191,192,193] and impedimetric [11,91,180,181,182,183,184,185,187,188,190,191,194,195] electrochemical sensor systems have been more commonly used for determination of abused drugs. The results from the literature survey are summarized in Table S3.

Amperometric techniques are commonly used due to high sensitivity and wide linear range and have different measurement modes, such as chronoamperometry, cyclic voltammetry (CV), and differential pulse voltammetry (DPV) [139,196]. DPV and CV methods have been one of the most widely used techniques for the determination of abused drugs. In the CV method, a varying potential is applied to the working electrode, and while the potential in the forward direction is terminated, the potential in the reverse direction is started scanning. This method provides information about whether the reaction taking place in the working electrode is reversible [197]. DPV is a technique where an increased potential is applied to the system, and the current is measured before and after the pulse. The obtained difference in the peaks is directly proportional to the concentration of target molecules [197]. In the impedimetric biosensors, electrochemical impedance spectroscopy (EIS) is used for measurements. EIS is a technique for determining electrode kinetics and binding characteristics of the analyte on the electrode surface with the application of an alternative current at different frequencies to the system [198]. As a result of the measurements, electrochemical spectrum data are obtained, and the impedance values are calculated accordingly [199,200].

In the last twenty years, electrochemical aptasensors have stood out as the most commonly used technique for the determination of abused drugs. Different electrodes types, such as gold [179,182,187,192,194,195], carbon [11,178,180,181,184,185,188,189,190,191,193] and an indium tin oxide, array electrodes [186] were used. Carbon electrodes are commonly used for sensors application due to advantageous properties, such as their reasonable costs, good mechanical hardness, high electrochemical activity, wide potential range, low background current, easy preparing steps, and replaceability [201]. A gold electrode is generally used as the working electrode in biosensor applications due to its biocompatibility, ease of binding of thiol groups, and useful electronic and optical properties [202,203,204]. Using this feature, recognition molecules, such as aptamers and antibodies, can be easily attached with thiol groups onto the gold electrode surface without pretreatments or any pre-modifications. Whereas for carbon electrodes, a pre-modification step with different coating materials, such as polymers [11,91,184,188,189,191] or nanostructures [181,190], is usually required to immobilize recognition molecules on the electrode surface.

Roushani et al. used a carbon electrode in three different works for cocaine detection, and they modified the surface of the electrode using multiwalled carbon nanotubes [180], ionic liquid [185], and a chitosan nanocomposite [178]. In these works, AuNPs [185], silver nanoparticles (AgNPs) [178], and PtNPs [180] were attached using terephthalate with thiol groups of cocaine aptamers. In the study with AgNPs, as a first, riboflavin was used as a redox probe, while the other studies with AuNPs and PtNPs were conducted with ferricyanide and significant differences were observed with the addition of cocaine in CV, DPV, and EIS peaks. Obtained aptasensor systems were successfully applied in the serum sample. With these aptasensors, low LOD values were achieved as follows: 100 pM, 150 pM, and 11 µM for the systems using PtNPs, AgNPs, and AuNPs, respectively.

Due to properties such as low-cost, easy integration, and lack of pretreatment need, an indium tin oxide array electrode was used for cocaine determination instead of gold and carbon electrodes [186]. The electrode surface was formed with multi-layer ferrocene appended poly(ethyleneimine), and the surface was then covered with AuNPs for binding of a thiol terminated cocaine aptamer. When a thrombin binding cocaine aptamer (C1) and cocaine were added to the environment, the C1 aptamer and SH-C2 aptamer were hybridized to bind cocaine, and thus CV and DPV peaks were significantly decreased. The designed aptasensor system successfully determined cocaine in plasma, serum, saliva, and urine samples.

In addition to the modification of the electrode surface, nanostructures were also used for the enhancement of CV and DPV peak signals in electrochemical aptasensor applications. Taghdisi et al. [179] used a gold electrode for cocaine detection by immobilizing the complementary strand of the aptamer (CS) on the electrode surface and conjugating it with the cocaine aptamer and synthesized single-walled carbon nanotubes (SWNTs) on the electrode surface. Cocaine analytes were prepared with different concentrations and were added on the modified electrode surface. This aptasensor platform was designed based on strong interaction between SWNTs and CS but very weak interaction between SWNTs and an aptamer-CS conjugate. In the absence of cocaine analyte, when SWNTs were added on the modified electrode with the aptamer-CS, CV peaks were decreased. However, in the presence of cocaine on the electrode surface, the aptamer was separated from the CS and was bonded with cocaine, while SWNTs were strongly connected to CS and CV peaks were increased. This aptasensor platform was successfully used for the determination of cocaine in serum samples. In a study by Xiong et al. [194], an electrochemical aptasensor was designed for codeine determination with surface-modified ZnS nanoparticles with β-cyclodextrin (ZnS-CD) which were synthesized for the production of the electrochemical signal. A 15-base codeine aptamer was attached on the gold electrode surface, and ZnS-CD added on the aptamer modified the electrode surface, binding from the CD part of the conjugate. The addition of the codeine analyte caused a change in the conformation of the aptamer and ZnS-CD was eliminated from the surface causing a change in the DPV peaks. In another recent study conducted by Su et al. [183], a gold electrode surface was coated with two-dimensional (2D) zirconium-based metal-organic framework nanosheets embedded with Au nanoclusters (denoted as 2D AuNCs@521-MOF) which has high physicochemical stability, high specific surface area, good electrochemical activity, and strong bio-affinity against phosphate groups. EIS, DPV, and CV measurements were performed, and LOD value was determined as 1.29 pM with EIS technique and 2.22 pM with DPV technique. Additionally, this aptasensor system was successfully used to determine cocaine in serum, urine, and saliva samples.

**Figure 8:**
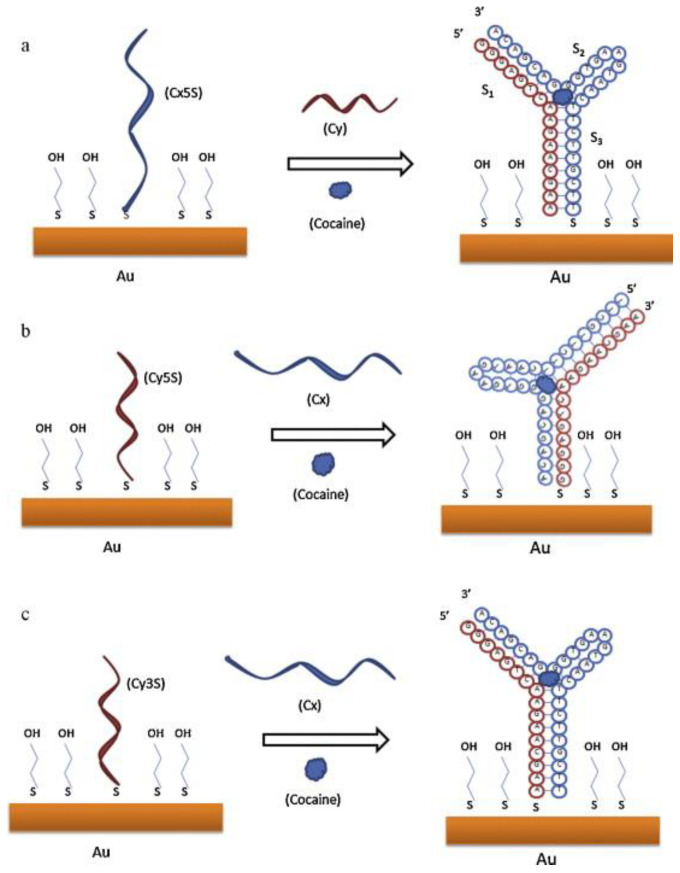
Aptasensor systems designing with fragments on Au surface with MCE as (**a**) Au/Cx5S/MCE, (**b**) Au/Cy5S/MCE, (**c**) Au/Cy3S/MCE. Reproduced with the permission of [183]. Copyright 2012 Published by Elsevier B.V.

Connecting the aptamer in its correct conformation on the electrode surface is crucial for the measurements in electrochemical aptasensor platforms. According to a study by Zhang et al. [194], a synthesized cocaine aptamer was divided into two fragments, and these fragments were called Cx and Cy. 3’ and 5’. Terminates of these fragments were modified with thiol groups, and three different aptasensor systems were designed with these fragments on the gold electrode (Au) surface with mercaptoethanol (MCE) as Au/Cx5S/MCE, Au/Cy3S/MCE, and Au/Cy5S/MCE. The Cy aptamer and cocaine were added into the Au/Cx5S/MCE system, while Cx and cocaine were added into the Au/Cy3S/MCE and Au/Cy5S/MCE systems. Impedance measurements of these systems were conducted in a ferricyanide redox probe solution, and the best results were obtained with Au/Cx5S/MCE modification in response to suitable immobilization of the aptamer and conformation of the supramolecular complex (Figure 8). In another study conducted by Shen et al. for the detection of cocaine, a similar strategy was followed, and the aptamer was divided into two fragments: Co3S and Co3B [187]. The Co3S fragment was attached on the gold electrode via a thiol group, and 3’ terminal of Co3B was labeled with biotin. In the presence of cocaine, the fragments were joined to capture the analyte. Then, streptavidin was added onto the electrode surface and was attached on the biotin terminal of the Co3B fragment, producing single-strand DNAs with rolling circle amplification in the presence of nucleotides and phi29 DNA polymerase. An important amplification for the determination of cocaine was achieved with streptavidin-alkaline phosphatase (ST-AP) and α-naphthyl phosphate (α-NP) addition to the surface. The LOD value of suggested aptasensor system was determined as 1.3 nM, and this platform was successfully used for cocaine detection in urine samples.

## 4. Discussion and Conclusions

Due to many advantageous properties, such as small sample volume, fast response, and low-cost, aptamer-based POC diagnostics platforms for abused drug detection have continued to develop since the beginning of the 21st century. Practical applications of aptasensors in forensic samples are highly dependent on selectivity and specificity of aptamers. As described in Section 2.3, aptamers have been selected in buffer media, by excluding the presence of proteins or other biological molecules. It should also be considered that aptamer–target interactions may be affected by the changes in pH and ion concentration. Therefore, to maximize the practical applications in real samples, body fluid composition should be mimicked, and SELEX buffer should be adjusted according to the forensic sample of interest. In addition, counter-SELEX can be performed against not only the structurally similar non-target molecules but also the biological samples obtained from healthy control individuals. Thereby, aptamer sequences that bind to other biological components in the sample can be eliminated. Incorporation of counter-SELEX may also allow direct sample analysis, possibly by excluding the need for a pre-treatment process. The possibility to adjust aptamer binding conditions provides flexibility in the selection of aptamers. The effect of the biological matrix can also be eliminated by post-SELEX optimization, as in the case of a cocaine-binding aptamer [87]. As shown in Table S1, the Kd values of cocaine, codeine, and ephedrine aptamers are in the micromolar range, indicating that aptamers have lower affinity than antibodies. As described in Section 2.2, the lower affinity of aptamers can be attributed to the technical challenges associated with the small chemical structures of abused drugs. As reviewed elsewhere [34,35], the SELEX method has been modified in different ways to enhance the small-molecule aptamer selection process. Similar strategies can be applied to develop high-affinity aptamers against abused drugs.

In this review, a bottom-up approach was followed from designing a custom-tailored aptamer synthesis to aptasensor development. Many optical and electrochemical aptasensor platforms have been reported previously in the literature. However, the majority of these aptasensors were focused on the detection of cocaine, leaving out other typical abused drugs, such as morphine, synthetic cannabinoids, and tetrahydrocannabinol (THC). In addition, these aptasensor platforms were all facilitated with synthetic samples spiked with analytes and have not yet been implemented on real human samples. This is due to two main reasons: low concentrations of some analytes in biological samples and complex environments of real samples interfering with detection and resulting in deviating signals [118]. To overcome these situations, pre-treatment steps, such as filtration [205], centrifugation [121], and dilution [206], are generally performed on real samples for other analytical techniques. In the forensic field, filtration and centrifugation techniques can be used to in aptasensors using real samples, such as urine, saliva, serum, sweat. Thus, not only the binding of the aptamer to the target drug is facilitated but also the concentration of the target molecule in the medium can be increased. In the recent abused drug detection studies, hair samples were also used as a real sample, and the determination of abused drugs in these samples is based on a certain length of hair [207]. To analyze with aptasensors, these samples can be pre-extracted in suitable environments. Thus, it can be concluded that efforts have been made to improve the sensitivity and selectivity of aptasensors to work with real biological samples. 

In addition, electrochemical aptasensor platforms were more prominent. Optical aptasensor platforms can now be considered as open research to be developed for the identification of all abused drugs. LFAs technologies have become one of the most important techniques due to many beneficial properties, such as enabling analysis outside laboratory environments, advanced devices for measurement are not needed, small sample volume, and ease of handling, but have not yet reached the place they deserve in the determination of abused drugs. Future studies should bear these points in mind. Additionally, these sensing platforms have recently been integrated into smartphones with mobile applications as diagnostic tools suitable for resource-limited settings, e.g., roadside measurements [145]. To both lower the cost and dependence on expert scientists, smartphone integration arises as a potential future diagnostic tool available for everyone, creating a bridge between controlled laboratory settings and chemical measurements [139].

To conclude, the pros and cons of these platforms are evaluated in Table 1, where a general SWOT analysis on the aptasensor for abused drugs is presented. 

## Figures and Tables

**Table 1:** SWOT analysis on aptasensors for abused drug detection.

Strengths (S)	Weaknesses (W)	Opportunities (O)	Threats (T)
Low-cost and widely applicable	Requirement of advanced devices	POC diagnosis of abused drug	In-laboratory, controlled environment testing
Sensitive and rapid	Requirement of expert scientists	On-site applications	Rapidly changing technologies
Simple and user-friendly	Lack of real-life sample studies	Real-time measurements	Difficulty in standardization
Adaptation possibilities for the newly designed drugs	Decision on choosing target analytes	Adjusting selectivity towards either a target analyte or a target group	Rapidly changing chemical formulations of designed drugs

## Supplementary Materials

The following are available online at https://www.mdpi.com/2079-6374/9/4/118/s1, Table S1: Binding affinity and post-SELEX optimization of aptamers selected for abused drugs, Table S2: Literature survey of optical aptasensors for abused drugs, Table S3: Literature survey of electrochemical aptasensors for abused drugs.
